# Maternal Haemoglobin and Short-Term Neonatal Outcome in Preterm Neonates

**DOI:** 10.1371/journal.pone.0089530

**Published:** 2014-02-25

**Authors:** Elodie Savajols, Antoine Burguet, Marianne Grimaldi, Florence Godoy, Paul Sagot, Denis S. Semama

**Affiliations:** 1 Department of Paediatrics, Centre Hospitalier Universitaire, Dijon, France; 2 Department of Obstetrics, Centre Hospitalier Universitaire, Dijon, France; Université de Montréal, Canada

## Abstract

**Objective:**

To determine whether there is a significant association between maternal haemoglobin measured before delivery and short-term neonatal outcome in very preterm neonates.

**Study design:**

We included prospectively all live births occurring from 25 to 32+6 weeks of gestation in a tertiary care centre between January 1^st^ 2009 and December 31^st^ 2011. Outborn infants and infants presenting with lethal malformations were excluded. Three hundred and thirty-nine mothers and 409 infants met the inclusion criteria. For each mother-infant pair a prospective record of epidemiologic data was performed and maternal haemoglobin concentration recorded within 24 hours before delivery was retrospectively researched. Maternal haemoglobin was divided into quartiles with the second and the third one regarded as reference as they were composed of normal haemoglobin values. Short-term outcome was defined as poor in case of death during hospital stay and/or grades III/IV intraventricular haemorrhage and/or periventricular leukomalacia and/or necessity of ventriculoperitoneal shunt.

**Results:**

The global rate of poor short-term neonatal outcome was 11.4% and was significantly associated with low maternal haemoglobin values. This association remained significant after adjustment for antenatal corticosteroids therapy, gestational age, parity, mechanism of preterm birth, mode of delivery and birth weight (aOR = 2.97 CI 95% [1.36–6.47]). There was no relation between short-term neonatal outcome and high maternal haemoglobin concentration values.

**Conclusion:**

We show that low maternal haemoglobin concentration at delivery is an independent risk factor for poor short-term neonatal outcome in very preterm neonates. This study is one of the first to show such an association within the preterm population.

## Introduction

Preterm delivery accounts for 75% of perinatal mortality and more than half of long-term morbidity.[1] Its overall incidence keeps increasing, especially in very preterm infants (born before 33 weeks of gestation) who present the poorest outcomes and the highest mortality rates.[1]–[3]

Several authors have reported a potential association between maternal haemoglobin concentration and various neonatal outcomes in the whole neonatal population. Described associations usually follow a U-shaped distribution with adverse outcomes at both ends of the haemoglobin range. The main underlying mechanisms involved are preterm birth and low birth weight.[4]–[12]

We decided to check if this association remained in very preterm neonates born before 33 weeks of gestation. We tested this hypothesis in a hospital based observational study with prospective data collection.

### Patients and Methods

This study was approved by our institutional review board (Comité de Protection des Personnes Est I, Bourgogne, France). After deliberation, the board decided that no written consent was needed.

We included prospectively all live births from 25 to 32weeks + 6 days of gestation that occurred in the university-based tertiary care centre in Dijon (Burgundy, France) between January 1^st^ 2009 and December 31^st^ 2011. All infants were hospitalized in our neonatal intensive care unit after primary care in the delivery room.

Outborn infants were excluded as maternal haemoglobin concentration values were usually unavailable in those cases. Inborn infants with missing data for maternal haemoglobin and infants presenting with lethal malformations (oesophageal atresia, diaphragmatic hernia, pulmonary hypoplasia and major congenital heart diseases) were also excluded.

For each mother-infant pair, a prospective and continuous record of epidemiologic data is routinely performed within our perinatal network since 1998.

In addition, for each birth, we retrospectively reported both the maternal haemoglobin concentration value and the maternal mean cell volume (MCV) recorded within the 24 hours before delivery. The recorded data were divided into quartiles (Q) with Q2 and Q3 clustered together and regarded as reference.

Our main outcome was poor short term neonatal outcome defined as: death during hospital stay and/or grades III–IV intraventricular haemorrhage (defined as per the Papile classification)[13], and/or cystic periventricular leukomalacia and/or necessity of ventriculoperitoneal shunt for post hemorrhagic hydrocephaly.[14] Intraventricular haemorrhage and cystic periventricular leukomalacia were diagnosed using cerebral ultrasound scanning performed by senior paediatricians and/or senior radiologists (Philips Type IE33).

In order to examine poor short-term neonatal outcome in relation to maternal haemoglobin concentration, we defined potential confounding variables classified as follows. Maternal characteristics: maternal age at delivery (divided into ≤24 years, 25–34 years and ≥35 years), parity (nulliparous or multiparous), maternal pre-pregnancy body mass index (BMI, kg/m^2^) and maternal smoking (defined as one cigarette or more per day during pregnancy). Pregnancy characteristics: gestational age, foetal gender, number of foetus and mode of delivery. Pregnancy characteristics also included mechanism of preterm birth (divided into spontaneous labour with no premature rupture of membranes, maternal bleeding (usually occurring after blood sample collection), premature rupture of membranes (PROM), gestational hypertension and others) and antenatal corticosteroids therapy (defined as at least one dose of betamethasone before delivery). Neonatal characteristics: intubation in the delivery room, surfactant use, blood cells transfusion, patent ductus arteriosus, hemodynamic instability, necroticizing enterocolitis (as defined per the Bell classification)[15] and chronic lung disease (defined as ventilator and/or oxygen dependence at 36 weeks of post-conceptionnal age).

### Statistical analysis

Qualitative variables were expressed as percentage and quantitative variable were expressed as mean ± SD.

The analysis followed 4 steps. We first sought to determine the potential relations between the confounding variables and short-term neonatal outcome in bivariate analysis. We performed the same analysis studying this time the potential relations between the confounding variables and maternal haemoglobin concentration quartiles.

We then studied in bivariate analysis the association between maternal haemoglobin concentration and short-term neonatal outcome.

We finally studied in multivariate analysis the association between maternal haemoglobin concentration and short term poor outcome while adjusting on the potential confounding variables previously defined.

All analyses were performed using chi2 test, Fisher test, variance analysis and logistic regression when needed. When variance analysis could not be used (i.e. Bartlett's test for equal variance was statistically significant with p<0.05), Kruskal-Wallis test had to be used. P values <0.05 were considered statistically significant. All analyses were performed with STATA 9® software (StataCorp. 2005. *Stata Statistical Software: Release 9*. College Station, TX: StataCorp LP).

## Results

**Figure 1:**
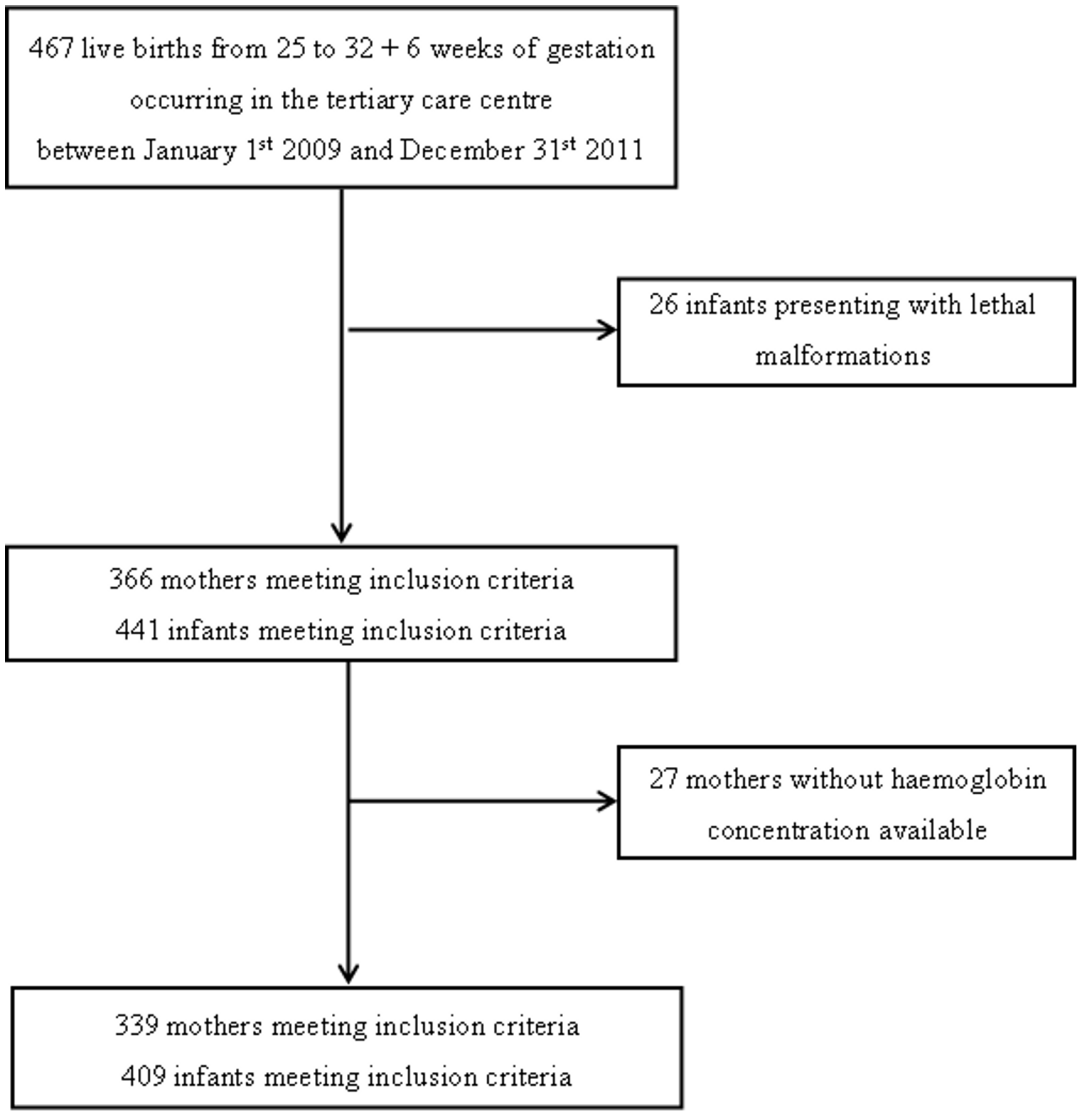
Flow Chart.

Four hundred and nine infants and 339 mothers met the inclusion criteria as represented in the flow chart (figure 1).

Maternal haemoglobin concentration values ranged from 7.6 g/dl to 10.7 g/dl within Q1 (n = 103), from 10.8 g/dl to 12.4 g/dl within Q2 and Q3 (n = 199) and from12.5 g/dl to 15.7 g/dl within Q4 (n = 107).

Associations between short-term neonatal outcome, maternal characteristics and pregnancy characteristics are shown in table 1. There were significantly more preterm neonates presenting with poor short-term outcome when they did not receive antenatal corticosteroids therapy and when their gestational age decreased.

**Table 1:** Short term neonatal outcome according to maternal and pregnancy characteristics.

		Poor short-term neonatal outcome		
	N infants	Yes	No	P value
	(n = 409)	(n = 48)	(n = 361)	
		%	%	
**Maternal age**		(46)	(357)	
15–24 years	(80)	12.5	87.5	
25–34 years	(248)	10.5	89.5	0.75
35–45 years	(75)	13.3	86.7	
**Parity**		(48)	(361)	
0	(219)	11.4	88.6	0.83
1 and more	(190)	12.1	87.9	
**Pre-pregnancy body mass index**		(30)	(307)	
<18.5	(23)	4.4	95.7	
18.5–24.9	(191)	7.9	92.2	0.41
25.0–45.0	(123)	11.4	88.6	
**Smoking**		(44)	(331)	
No	(269)	11.5	88.5	0.84
1 cigarette/day and more	(106)	12.3	87.7	
**Gestational age**		(48)	(361)	
25–27	(75)	29.3	70.7	
28–30	(163)	11.0	89.0	**0.001**
31–32 weeks	(171)	4.7	95.3	
**Foetal Gender**		(48)	(361)	
Boy	(213)	13.6	86.4	0.23
Girl	(194)	9.7	90.3	
**Multiple pregnancy**		(48)	(361)	
Yes	(140)	8.6	91.4	0.15
No	(269)	13.4	86.6	
**Mechanism of preterm birth**		(48)	(361)	
Spontaneous labour with no PROM	(126)	11.9	88.1	
Maternal bleeding	(29)	17.2	82.8	
PROM	(133)	11.3	88.7	0.77
Gestational hypertension	(79)	12.7	87.3	
Others	(42)	7.1	92.9	
**Mode of delivery**		(48)	(361)	
Vaginal	(119)	10.1	89.9	0.51
Caesarean	(290)	12.4	87.6	
**Antenatal corticosteroids therapy**		(47)	(361)	
No	(18)	33.3	66.7	**0.01**
Yes	(390)	10.5	89.5	

Associations between quartiles of maternal haemoglobin concentration values, maternal MCV, maternal characteristics and pregnancy characteristics are shown in table 2. A significant shift of haemoglobin concentrations towards lower values was observed in multiparous women when compared to nulliparous women. A similar shift was observed in women presenting with PROM, while women presenting with gestational hypertension had significantly higher haemoglobin concentration values when compared with other mechanisms of preterm birth.

**Table 2:** Quartiles (Q) of maternal haemoglobin concentration according to maternal and pregnancy characteristics.

	Quartiles of maternal haemoglobin concentration				
	Women	Q1	Q2–Q3	Q4	P
	(n = 339)	≤10.7 g/dl	10.8–12.4 g/dl	≥12.5/dl	
		(n = 85)	(n = 165)	(n = 89)	
		%	%	%	
**Maternal mean corpuscular volume**					
Mean (SD)		89.4 (7.9)	91.8 (5.6)	92.8 (5.1)	0.01
**Maternal age**		(83)	(164)	(87)	
15–24 years	(70)	30.0	41.4	28.6	
25–34 years	(199)	22.6	55.3	22.1	0.07
35–45 years	(65)	26.1	38.5	35.4	
**Parity**		(85)	(165)	(89)	
0	(177)	17.5	53.1	29.4	0.01
1 and more	(162)	33.3	43.8	22.8	
**Pre-pregnancy body mass index**		(67)	(138)	(74)	
<18.5	(18)	27.8	61.1	11.1	
18.5–24.9	(160)	23.7	53.7	22.5	0.07
25.0–45.0	(101)	23.8	43.6	35.6	
**Smoking**		(79)	(149)	(88)	
No	(218)	24.8	49.1	26.1	0.86
1 cigarette/day and more	(91)	27.5	46.1	26.4	
**Gestational age**		(85)	(165)	(89)	
25–27	(63)	28.6	47.6	23.8	
28–30	(143)	23.8	47.5	28.7	0.89
31–32 weeks	(133)	24.8	50.4	24.8	
**Multiple pregnancy**		(85)	(165)	(89)	
Yes	(70)	31.4	47.1	21.4	0.32
No	(269)	23.4	49.1	27.5	
**Mechanism of preterm birth**		(85)	(165)	(89)	
Spontaneous labour with no PROM	(98)	20.4	59.2	20.4	
Maternal bleeding	(26)	19.2	53.8	26.9	
PROM	(103)	43.7	44.7	11.6	0.001
Gestational hypertension	(77)	6.5	42.9	50.6	
Others	(35)	28.6	40.0	31.4	
**Mode of delivery**		(85)	(165)	(89)	
Vaginal	(97)	21.6	60.8	17.5	0.01
Caesarean	(242)	26.4	43.8	29.7	
**Antenatal corticosteroids therapy**		(84)	(165)	(89)	
No	(15)	20.0	53.3	26.7	0.89
Yes	(323)	25.1	48.6	26.3	

Infants whose mothers had high haemoglobin concentration values (Q4) had a significantly lower mean birth weight than the ones whose mothers had haemoglobin concentration values within Q1 or Q2–Q3 (1184±383 g, 1378±389 g, 1353±379 g respectively; p = 0.001).

There was no statistically significant association between maternal haemoglobin concentration values and rates of intubation in the delivery room, surfactant use, need for blood transfusion, patent ductus arteriosus, hemodynamic instability, necrotizing enterocolitis and chronic lung disease.

The global rate of poor short-term neonatal outcome was 11.4%. There was a statistically significant association between maternal haemoglobin concentration values and short-term neonatal outcome with an increased rate of poor outcome in infants whose mothers had low haemoglobin concentration values (Q1). This association remained significant after adjustment for antenatal corticosteroids therapy, gestational age, parity, mechanism of preterm birth, mode of delivery and birth weight (table 3). There was no statistically significant relation between short-term neonatal outcome and high maternal haemoglobin concentration values, before or after adjustment for the confounding variables.

**Table 3:** Relation between short term neonatal outcome and maternal hemoglobin concentration.

		Poor short term outcome						
	n	Yes	No	OR [CI 95%]	aOR^1^ [CI 95%]	aOR^2^[CI 95%]	aOR^3^[CI 95%]	aOR^4^[CI 95%]
	(409)	(n = 48)	(n = 361)					
		%	%					
**Q1**≤10.7 g/dl	(103)	(20)	(83)	2.42 [1.22–4.82]	2.55 [1.24–5.25]	2.99 [1.37–6.53]	2.97 [1.36–6.47]	2.97 [1.36–6.47]
		19.4	80.6					
**Q2–3**10.8–12.4 g/dl	(199)	(18)	(181)	1.0	1.0	1.0	1.0	1.0
		9.0	91.0					
**Q4**≥12.5/dl	(107)	(10)	(97)	1.04 [0.44–2.43]	0.82 [0.35–1.93]	0.80 [0.33–1.94]	0.78 [0.31–1.98]	0.78 [0.31–1.98]
		9.3	90.7					

aOR^1^: adjustment for gestational age, birth weight.

aOR^2^: adjustment for gestational age, birth weight, mechanism of preterm birth.

aOR^3^: adjustment for gestational age, birth weight, mechanism of preterm birth, antenatal corticosteroids therapy.

aOR^4^: adjustment for gestational age, birth weight, mechanism of preterm birth, antenatal corticosteroids therapy, maternal age, parity, mode of delivery.

**Figure 2:**
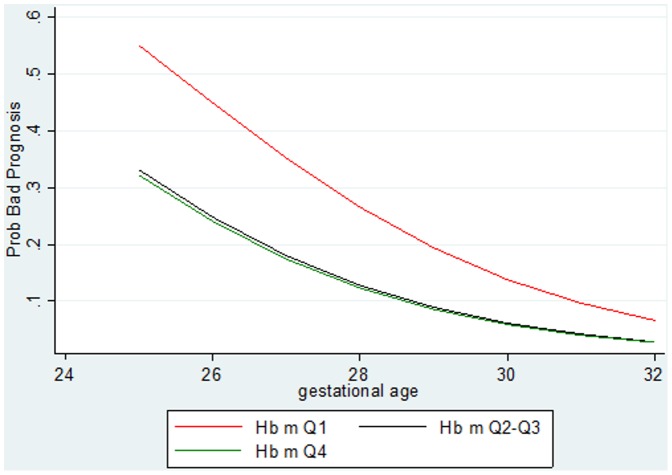
Probability of poor short-term neonatal outcome in relation to gestational age and maternal haemoglobin quartile (Hb m Q). The results are adjusted for birth weight, mechanism of preterm birth, antenatal corticosteroid therapy, maternal age, parity and mode of delivery.

The association between poor short-term neonatal outcome, gestational age and maternal haemoglobin concentration value is shown in figure 2. After adjusting for birth weight, mechanism of preterm birth, antenatal corticosteroid therapy, maternal age, parity and mode of delivery, infants whose mothers had a haemoglobin concentration value within Q1 had a poorer short-term neonatal outcome, whatever their gestational age. No difference was found between other quartiles.

## Discussion

This observational study showed that neonates born before 33 weeks of gestation had a poorer short-term neonatal outcome when prenatal maternal haemoglobin concentration was low. This association remained strong and significant after adjustment for gestational age, birth weight, mechanism of preterm birth, antenatal corticosteroids therapy, maternal age, parity and mode of delivery. To our knowledge, our study is the first to focus on the potential impact of maternal haemoglobin concentration on very preterm neonates. It is also to be noted that the upper limit of Q1 concurs with the definition of anaemia in pregnancy by World Health Organization.[16]

The association between maternal haemoglobin and neonatal outcomes has already been studied in the general population of neonates and seems to follow a U-shaped distribution with adverse outcomes at both ends of the haemoglobin range largely mediated through preterm birth and low birth weight.[7], [8], [17] However, as our study was dealing only with preterm neonates, the effect of preterm birth could not be taken into account. In our very preterm population, we did not find a U-shaped distribution: preterm neonates whose mothers had low haemoglobin concentration value (Q1) had a poorer short-term outcome but no difference was found in terms of outcome in infants whose mothers had the highest haemoglobin concentration value (Q4). This absence of difference persisted after adjusting for gestational age and birth weight. The effect of low maternal haemoglobin concentration was then neither mediated through associations with gestational age nor with birth weight in our study. Other factors were probably involved.

A deeper analysis of our results could lead to question a potential association between maternal haemoglobin concentration and mechanisms of preterm birth. At the higher end of haemoglobin values range (Q4), we find both a significantly higher proportion of women presenting with preeclampsia and a significantly higher proportion of neonates presenting with a lower birth weight. It is known that the ability to produce a large increase in plasma volume between the first and the second pregnancy trimester is one of the hallmarks of successful pregnancy as it is probably linked with correct uterine perfusion. Failure to produce this expansion may lead to insufficient uterine perfusion and thus to preeclampsia and intra uterine growth restriction.[18] Moreover, women not undergoing this expansion are bound to present a higher haemoglobin concentration due to hemoconcentration. At the other end of haemoglobin values range, we find a potential association between a lower haemoglobin value and a higher rate of preterm rupture of the membranes. This association has already been described in the literature while the physiopathological mechanisms underlying it still remain unclear. However, in spite of those potential associations between maternal haemoglobin concentration and mechanism of preterm birth at both ends of the haemoglobin values range, the association between maternal haemoglobin concentration and short-term neonatal outcome remains significant after adjusting on those very mechanisms of preterm birth. Consequently, this association cannot be explained only through the mechanisms of preterm birth. Other possible physiopathological mechanisms could be functional impairments at tissue level created by low haemoglobin concentration [19], [20] or increased oxidative stress due to iron deficiency [21], [22], which is one of the major mechanisms underlying low maternal haemoglobin concentration during pregnancy.[16] Those tissue functional impairments and/or oxidative damages, should they take place within the foeto-placental unit, could result in a poorer neonatal outcome.Further research will be needed to confirm those hypotheses.
