# Correction: Maternal Haemoglobin and Short-Term Neonatal Outcome in Preterm Neonates

**DOI:** 10.1371/journal.pone.0179964

**Published:** 2017-06-14

**Authors:** Elodie Savajols, Antoine Burguet, Marianne Grimaldi, Florence Godoy, Paul Sagot, Denis S. Semama

The following information is missing from the Competing Interests section: Author PS received funding from the following commercial companies: Merck Serono, Finox Biotech, MSD France SAS, Teva Santé SAS, Allergan France, Gedeon Richter France, Effik S.A., Karl Storz Endoscopie France, GE Medical Systems SCS, Laboratoires Genevrier, H.A.C. Pharma, and Ipsen. The authors confirm that none of this funding was used to support the research in this study. There are no patents, products in development or marketed products to declare. This does not alter the authors’ adherence to all the PLOS ONE policies on sharing data and materials.
